# Pre-ICU hospital length of stay and 30-day mortality in the very old: a national cohort of 315 042 ICU admissions

**DOI:** 10.1016/j.aicoj.2026.100116

**Published:** 2026-07-16

**Authors:** Björn Ahlström, Miklos Lipcsey

**Affiliations:** a Anesthesiology and Intensive Care, Department of Surgical Sciences, Uppsala University, Uppsala, Sweden; b Center for Clinical Research Dalarna, Uppsala University, Sweden; c Hedenstierna Laboratory, Department of Surgical Sciences, Uppsala University, Uppsala, Sweden

**Keywords:** Intensive care, Age, Mortality, Risk factors, Time in hospital

## Abstract

**Background:**

The number of very old (≥80 years) patients (VIPs) admitted to intensive care units (ICUs) is rising. Hospital length of stay before ICU admission (pre-ICU LOS) is associated with short-term mortality, but whether this association is uniform across adult age is unclear. We examined how age modifies the association between pre-ICU LOS and 30-day mortality.

**Methods:**

We included all adult first ICU admissions in the Swedish Intensive Care Registry 2005–2016, with comorbidity and vital status from national registers. We fit a logistic regression on 30-day mortality with restricted cubic splines for age, pre-ICU LOS, and Charlson comorbidity index, with interactions. Marginal predicted risks and within- and between-age risk differences (RDs) were from the model. Two sensitivity analyses included Simplified Acute Physiology Score (SAPS3) functional form of pre-ICU LOS and age, and adjustment for acute physiology at admission (SAPS3 Box III) with multiple imputation.

**Results:**

Among 315 042 patients, 30-day mortality rose with pre-ICU LOS in every age decile, but the magnitude of this gradient varied with age. In VIPs, the within-age RD from day 0 to day 60 was 12.8 percentage points (95% CI 10.0–15.6) against a day-0 baseline of 34.6 %, compared with 17.5 (16.0–18.9) in 60–69-year-olds. Findings were consistent across sensitivity analyses.

**Conclusions:**

Pre-ICU hospital length of stay is a weaker prognostic marker in VIPs than in the majority of ICU patients, although the extent to which this reflects biology versus age-dependent ICU triage remains unresolved.

ClinicalTrials.gov identifier NCT07571213 (retrospectively registered).

## Highlights

• National Swedish cohort (n = 315 042) reveals age-dependent pre-ICU LOS effect.
• The mortality gradient varies with age; ≥80-year-olds show flatter slopes.
• Pre-ICU stay is a weaker prognostic marker in VIPs than in most ICU patients.
• G-computation yields absolute risks, avoiding the pitfalls of hazard ratios.
• Age-dependent ICU triage may partly drive the attenuation in the very old.

## Graphical abstract

**Figure:**
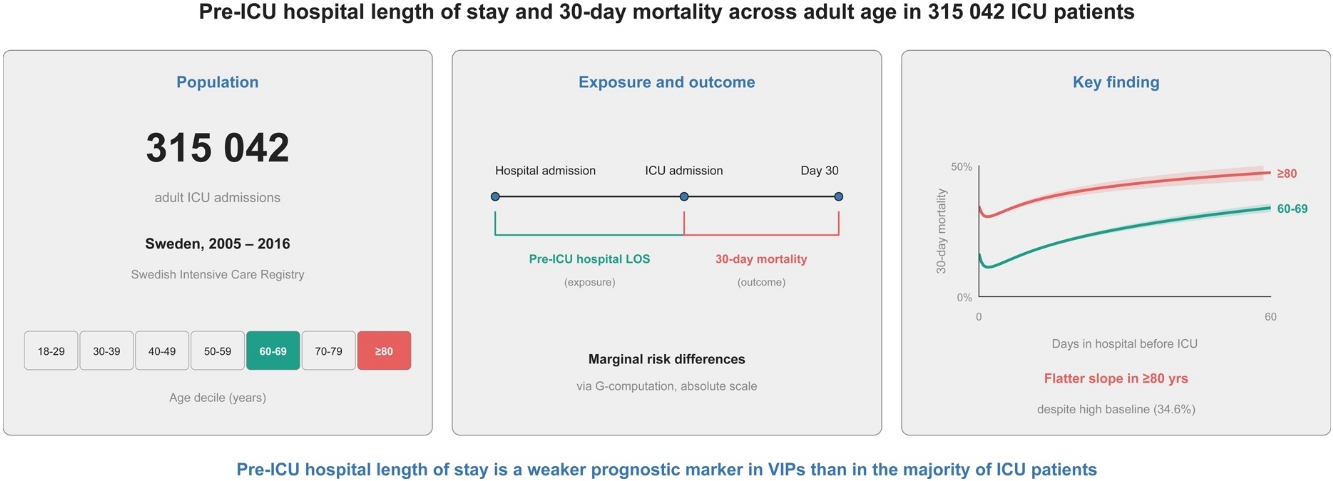

## Introduction

Due to global demographic changes, the number of very old patients (≥80 years) assessed for intensive care unit (ICU) admission because of impending or manifest organ failure is rising, and is expected to continue rising [1,2]. ICU care is demanding on the patient, often painful and stressful [3]. Very old ICU patients (VIPs), ≥80 years, are at higher risk of complications such as ICU-acquired delirium and weakness than younger patients [4,5], and, with lower physiological reserves, they are more prone to move from independent living before admission to dependence on others for activities of daily living afterwards [6]. These factors together make well-balanced decisions about ICU admission, and about limitations to care once in the ICU, particularly important in this age group [7].

The prognosis of the individual VIP in the ICU is shaped by pre-admission frailty and comorbidity as well as by the acute health deterioration that triggered admission [8]. Hospital length of stay before ICU admission is a prognostic factor associated with short-term mortality, but whether this association holds uniformly across the adult age range, or is aggravated or attenuated in VIPs, is not established [7,9,10]. We therefore examined how age modifies the association between pre-ICU hospital length of stay and short-term mortality in a national cohort of adult ICU patients.

## Methods

We conducted a national registry-based retrospective cohort study on prospectively collected data, approved by the Regional Ethics Committee of Uppsala (DNR 2016/421), which also waived informed consent. We followed the Declaration of Helsinki and report the study according to the RECORD extension of STROBE. The study was retrospectively registered with ClinicalTrials.gov (NCT07571213). We included all adult (≥18 years) patients with a first ICU admission reported to the Swedish Intensive Care Registry (SIR) between January 1, 2005 and December 31, 2016. SIR coverage of Swedish general ICUs rose from approximately 50% in 2005 to over 80% in 2010 and 100% by 2017 [11,12]. From SIR, we obtained demographics, admission route, case-mix, and SAPS3 with its components [10]. Comorbidities and in-patient care episodes came from the National Patient Registry (NPR)–Inpatient Part, and vital status from the Swedish Cause of Death Registry, both under statutory reporting to the National Board of Health and Welfare [13,14]. Records were linked via the Swedish personal identification number; patients without a valid number had been excluded before data delivery [15].

### Exposure

Pre-ICU hospital length of stay (pre-ICU LOS) was the number of full days between hospital and ICU admission (zero for direct-from-ER admissions). The primary source was the SAPS3 reporting field; where missing, we reconstructed it from NPR care episodes overlapping with or immediately adjacent to the ICU episode. In the primary model, pre-ICU LOS entered on the log scale as log (days + 1), which accommodates the large fraction of zero values and the long right tail (531 patients had 61–6703 days). In the categorical sensitivity analysis, we used the SAPS3 Box I form (0–13, 14–27, ≥28 days).

### Outcome

The outcome was all-cause 30-day mortality from ICU admission, as a binary endpoint. Follow-up was complete for all patients, with minimal expected emigration-related censoring during this window. Hospital and ICU discharge, therefore, constituted neither censoring nor a competing event.

### Descriptive analysis, conceptual framework and primary model

Baseline characteristics were summarized by age decile (18–29, 30–39, …, ≥80 years) using median [Q1–Q3] or n (%). Between-group differences were tested with the Kruskal–Wallis rank sum test and the χ^2^ test, with Dunn's test or the pairwise proportion test (Bonferroni-corrected) for *post-hoc* comparisons. The adjustment set was chosen *a priori* from a directed acyclic graph (Additional file, Figure S1). The primary model was a binary logistic regression on 30-day mortality with age, pre-ICU LOS and Quan’s updated Charlson comorbidity index (CCI) [16] modelled as restricted cubic splines, sex as a binary factor, and the three pairwise interactions (age × pre-ICU LOS, pre-ICU LOS × sex, and age × sex) encoded as products of the corresponding spline bases (or spline-by-factor for sex) [17]. From this model, we derived, by G-computation [18], (i) the marginal predicted probability of 30-day mortality for each age decile; (ii) within-age risk differences (RDs) relative to day 0; and (iii) between-age RDs relative to the 18–29 reference. The estimation procedure, and the model's discrimination and calibration, are described in the Additional file (Supplementary statistical methods; Figure S2, Table S1). SAPS3 Box III was not included in the primary adjustment set because it represents acute physiology measured after the exposure window and is a plausible mediator of the pre-ICU LOS effect on mortality; it was instead examined in a sensitivity analysis. Point estimates are reported with a 95% confidence interval in parentheses.

### Missing data

All variables in the primary adjustment set were complete after the SAPS3-plus-NPR reconstruction of pre-ICU LOS (N = 315 042). SAPS3 Box III, used only in the sensitivity analysis, was missing in approximately 39% of patients and imputed by multivariate imputation by chained equations [19]. Imputation diagnostics are shown in Additional file, Figures S3 and S4.

### Sensitivity analyses

We performed two sensitivity analyses, the first to test robustness to the choice of functional form for age and pre-ICU LOS, and the second to examine whether the primary association persisted once acute physiology at admission was held fixed. In the first, continuous age and pre-ICU LOS were replaced by their SAPS3 Box I form (age: <40, 40–59, 60–69, 70–74, 75–79, ≥80; pre-ICU LOS: 0–13, 14–27, ≥28 days), keeping sex, CCI and all pairwise interactions. In the second, we added SAPS3 Box III with restricted cubic splines to the primary model.

### Software

All analyses used R 4.5.1 (R Core Team 2024) with R packages according to Additional file, Table S2. Two-sided p-values < 0.05 were considered statistically significant.

## Results

Of 315 042 adult first ICU admissions from 2005 to 2016, median age was 65 years [IQR 50–76], 41.6% were women, and 51.3% arrived from the emergency department (Table 1). 30-day mortality was 16.2% overall and rose monotonically across age deciles, from 2.0% in 18–29-year-olds to 33.8% in patients ≥80 (Table 2).

**Table 1:** Baseline characteristics by age decile.

	18−29	30−39	40−49	50−59	60−69	70−79	≥80	Overall	p-value
N	29 732	20 758	27 107	41 511	70 875	74 950	50 109	315 042	
Age	23.0 [20.0−26.0]	35.0 [32.0−37.0]	45.0 [42.0−47.0]	55.0 [52.0−57.0]	65.0 [62.0−67.0]	74.0 [72.0−77.0]	84.0 [81.0−87.0]	65.0 [50.0−76.0]	<0.001
Female sex	13 497 (45.4%)	10 284 (49.5%)	11 142 (41.1%)	15 408 (37.1%)	25 679 (36.2%)	30 216 (40.3%)	24 913 (49.7%)	131 139 (41.6%)	<0.001
Hospital Type									
County	13 650 (45.9%)	9492 (45.7%)	11 013 (40.6%)	15 820 (38.1%)	26 550 (37.5%)	29 130 (38.9%)	22 322 (44.5%)	127 977 (40.6%)	<0.001
District	7496 (25.2%)	4238 (20.4%)	5776 (21.3%)	7543 (18.2%)	12 456 (17.6%)	14 479 (19.3%)	13 595 (27.1%)	65 583 (20.8%)	
University	8586 (28.9%)	7028 (33.9%)	10 318 (38.1%)	18 148 (43.7%)	31 869 (45.0%)	31 341 (41.8%)	14 192 (28.3%)	121 482 (38.6%)	
Admitted from ER	14 645 (74.1%)	7818 (60.7%)	10 384 (62.7%)	13 276 (55.5%)	19 220 (46.1%)	18 647 (41.6%)	14 695 (44.9%)	98 685 (51.3%)	<0.001
SAPS3	36.0 [32.0−43.0]	37.0 [32.0−45.0]	44.0 [37.0−53.0]	46.0 [39.0−57.0]	53.0 [44.0−64.0]	59.0 [50.0−70.0]	64.0 [55.0−73.0]	52.0 [41.0−65.0]	<0.001
Missing	10 080 (33.9%)	7945 (38.3%)	10 652 (39.3%)	17 716 (42.7%)	29 434 (41.5%)	30 380 (40.5%)	17 532 (35.0%)	123 739 (39.3%)	
Surgical admission	3804 (13.0%)	4047 (20.3%)	4065 (16.3%)	6432 (18.1%)	12 430 (21.4%)	13 854 (22.5%)	9600 (20.7%)	54 232 (19.7%)	<0.001
Missing	494 (1.7%)	835 (4.0%)	2154 (7.9%)	6021 (14.5%)	12 669 (17.9%)	13 504 (18.0%)	3720 (7.4%)	39 397 (12.5%)	
CCI	0.0 [0.0−0.0]	0.0 [0.0−0.0]	0.0 [0.0−0.0]	0.0 [0.0−1.0]	0.0 [0.0−2.0]	0.0 [0.0−2.0]	1.0 [0.0−2.0]	0.0 [0.0−2.0]	<0.001
CRRT	209 (0.7%)	253 (1.2%)	572 (2.1%)	1128 (2.7%)	2449 (3.5%)	2651 (3.5%)	844 (1.7%)	8106 (2.6%)	<0.001
IMV	4447 (15.0%)	3740 (18.0%)	6169 (22.8%)	10 988 (26.5%)	21 091 (29.8%)	21 783 (29.1%)	10 461 (20.9%)	78 679 (25.0%)	<0.001

ER is Emergency Room.

SAPS3 is the Simplified Acute Physiology Score 3 [10].

CCI is the Quan updated Charlson comorbidity index [16].

CRRT is continuous renal replacement therapy.

IMV is invasive mechanical ventilation.

**Table 2:** Time in hospital before ICU admission and mortality, by age decile.

Age deciles	n (%)	Mean time in hospital before ICU (SD)	Median time in hospital before ICU admission [Q1-Q3]	30-day mortality n (%)	90-day mortality n (%)	6-month mortality n (%)	1-year mortality n (%)
18−29	29 732 (9.4)	1.2 (16.1)	0 [0−0]	597 (2.0%)	714 (2.4%)	817 (2.7%)	1008 (3.4%)
30−39	20 758 (6.6)	1.4 (13.4)	0 [0−1]	649 (3.1%)	812 (3.9%)	951 (4.6%)	1159 (5.6%)
40−49	27 107 (8.6)	2.0 (34.1)	0 [0−1]	1731 (6.4%)	2075 (7.7%)	2374 (8.8%)	2781 (10.3%)
50−59	41 511 (13.2)	2.3 (26.6)	0 [0−1]	4318 (10.4%)	5224 (12.6%)	5968 (14.4%)	7028 (16.9%)
60−69	70 875 (22.5)	2.7 (24.4)	1 [0−2]	10 647 (15.0%)	13 180 (18.6%)	14 865 (21.0%)	17 154 (24.2%)
70−79	74 950 (23.8)	2.8 (8.2)	1 [0−2]	16 178 (21.6%)	19 942 (26.6%)	22 332 (29.8%)	25 253 (33.7%)
≥80	50 109 (15.9)	2.2 (5.3)	0 [0−2]	16 921 (33.8%)	20 209 (40.3%)	22 398 (44.7%)	25 131 (50.2%)
Overall	315 042 (100.0)	2.3 (19.6)	0 [0−1]	51 041 (16.2%)	62 156 (19.7%)	69 705 (22.1%)	79 514 (25.2%)
			*p = <0.001	*p = <0.001	*p = <0.001	*p = <0.001	*p = <0.001

*p-value for the global null hypothesis of no difference across age groups (Kruskal–Wallis for continuous variables, chi-squared for categorical variables). Post-hoc Dunn’s test (Bonferroni adjusted) yielded p < 0.001 for difference between all groups regarding Median time in hospital before ICU admission and all mortality outcomes, reference ≥80. ICU is intensive care unit.

### Primary model

**Fig. 1:**
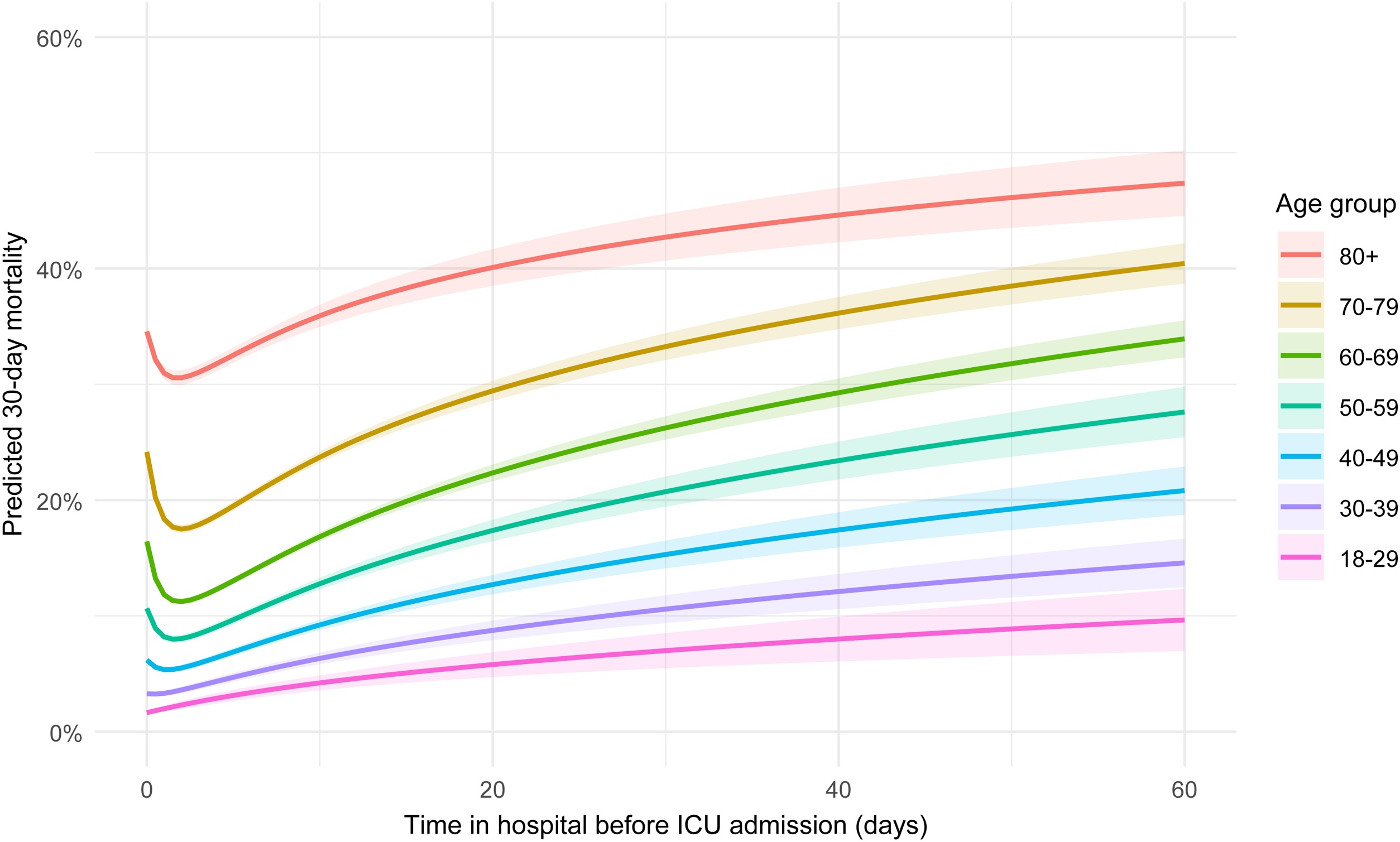
Marginal predicted 30-day mortality by age decile across pre-ICU hospital length of stay. Each line is the G-computation estimate obtained by fixing all patients within an age decile to the pre-ICU LOS value on the x-axis and averaging the predicted probabilities from the logistic model with restricted cubic splines; shaded bands are 95% percentile confidence intervals.

Marginal predicted 30-day mortality rose with pre-ICU LOS in every age decile, but with visibly different slopes, flattest in the youngest deciles and in patients ≥80 (Fig. 1).

Risk differences relative to day 0 showed the age-dependent effect of pre-ICU LOS (Table 3, Fig. 1). In the middle deciles, an initial decrease in predicted risk preceded the expected positive gradient. This dip was numerically deepest in 70–79-year-olds, reaching −5.8 percentage points (pp) (95 % CI −6.2 to −5.4) at day 1 and −6.3 pp (−6.8 to −5.8) at day 3 before returning toward zero by day 14. Day-60 risk differences were largest in the middle deciles (17.0 pp in 50–59, 17.5 pp in 60–69, 16.3 pp in 70–79-year-olds) and smaller at both ends of the age range, but asymmetrically: in 18–29-year-olds an RD of 8.0 pp built on a day-0 baseline of 1.6%, while in patients ≥80 an RD of 12.8 pp sat atop a day-0 baseline of 34.6%.

**Table 3:** Within-age risk differences (percentage points) for 30-day mortality across pre-ICU hospital length of stay, relative to day 0. Primary model.

Age decile	Day 1	Day 3	Day 7	Day 14	Day 28	Day 60
18−29	0.3 (0.1 to 0.6)	1.0 (0.5–1.4)	2.0 (1.4–2.6)	3.3 (2.5–4.2)	5.1 (3.8–6.7)	8.0 (5.6–10.9)
30−39	0.0 (−0.2 to 0.3)	0.7 (0.3–1.0)	2.1 (1.7–2.6)	4.1 (3.5–4.9)	7.0 (5.9–8.9)	11.3 (9.3–13.5)
40−49	−0.8 (−1.1 to −0.5)	−0.3 (−0.6 to 0.1)	1.7 (1.3–2.1)	4.6 (4.0–5.3)	8.6 (7.7–10.0)	14.6 (12.7–17.0)
50−59	−2.5 (−2.8 to −2.1)	−2.2 (−2.7 to −1.7)	0.3 (−0.2 to 0.9)	4.2 (3.5–4.9)	9.5 (8.4–10.7)	17.0 (15.0–19.2)
60−69	−4.6 (−4.9 to −4.3)	−4.8 (−5.2 to −4.4)	−1.8 (−2.2 to −1.4)	2.9 (2.3–3.5)	9.1 (8.2–10.0)	17.5 (16.0–18.9)
70−79	−5.8 (−6.2 to −5.4)	−6.3 (−6.8 to −5.8)	−2.9 (−3.4 to −2.3)	2.2 (1.5–2.9)	8.4 (7.4–9.5)	16.3 (14.6–17.9)
≥80	−3.6 (−4.3 to −2.9)	−3.5 (−4.4 to −2.7)	−0.6 (−1.5 to 0.3)	3.3 (2.0–4.7)	7.7 (5.7–9.7)	12.8 (10.0–15.6)

Values are risk differences in percentage points with 95% bootstrap percentile CI in parentheses, obtained by G-computation from a logistic model.

ICU is intensive care unit.

Between-age risk differences. At every pre-ICU LOS, older age was associated with substantially higher 30-day mortality (Fig. 1, Additional file, Table S3). The gap between ≥80 and 18–29-year-olds widened modestly from 32.9 pp at day 0–37.7 pp at day 60. Between ≥80 and middle-aged deciles, by contrast, the gap narrowed at longer stays (≥80 vs 50–59: 23.9 pp at day 0 vs 19.8 pp at day 60), reflecting the shallower within-age LOS gradient in the oldest group.

### Sensitivity analyses

Results were consistent across both sensitivity analyses. With age and pre-ICU LOS in their SAPS3 Box I categorical forms, within-age risk differences relative to 0–13 days were clearly positive in the youngest group (<40 years: 7.8 pp for 14–27 days, 8.4 pp for ≥28 days; 95% CIs excluding zero) but small and compatible with zero in patients ≥80 (1.5 pp and 3.3 pp, respectively; Additional file, Figure S5, Tables S4-S5). Adjusting additionally for SAPS3 Box III did not alter the overall age-dependent pattern (Additional file, Figure S6, Tables S6–S7), but the initial within-age dip was systematically attenuated in all affected deciles (70–79-year-olds at day 3: −4.6 pp [95% CI −5.2 to −4.0] vs. −6.3 pp in the primary model; sign change in ≥80 at day 7: 0.5 pp vs. −0.6 pp).

## Discussion

In this national cohort of 315 042 adult ICU patients, the association between pre-ICU hospital length of stay and 30-day mortality was strongly age-dependent. The predicted probability of death rose with pre-ICU LOS in every age decile, but the magnitude of this gradient varied with age, peaking in the middle deciles and attenuated in VIPs.

The overall positive association between longer pre-ICU stays and mortality is consistent with earlier reports [9,10] and with a recent meta-analysis of 34 observational studies, in which delayed ICU admission carried a pooled OR for mortality of 1.61 (1.44–1.81) [20]. That literature is heterogeneous, however, and much of the heterogeneity reflects differences in the outcome, the definition of delayed ICU admission, and the population studied. The exposure has been operationalized in two quite different ways. Some studies count the hours a patient spends waiting for an ICU bed, which largely indexes emergency-department throughput and ICU bed availability; a study defining delay as more than three hours from referral, for instance, found no difference in ICU stay or mortality after adjustment (OR 1.27, 0.81–2.0), although delayed patients required more respiratory support [21]. Others, including the present cohort, count the days spent on a hospital ward before ICU admission, which instead reflects ward-based deterioration and the progressive selection of who is ultimately admitted. Even among the latter, results vary with the outcome chosen. In 2,248 emergency admissions, a prolonged pre-ICU stay predicted hospital but not ICU mortality [22], and in a resource-limited setting, it was unrelated to ICU mortality (adjusted OR 1.00, 0.98–1.03) [23]. A single-center series [24] and a large registry analysis in patients with hematological malignancy, by contrast, found pre-ICU hospital LOS independently associated with higher mortality (OR 1.02, 1.01–1.02, by day) [25].

Two features distinguish the present analysis. First, our outcome is 30-day all-cause mortality. Unlike the ICU mortality used in several prior studies, it captures deaths occurring after ICU and hospital discharge and is therefore not distorted by differential discharge; it is also the endpoint Khan and colleagues specifically suggested as a worthwhile extension [23]. Second, earlier work has reported pre-ICU LOS as a single average effect across the adult ICU population without examining how it varies with age [23,24], leaving the prognostic weight of pre-ICU LOS specifically in VIPs (≥80 years) largely unaddressed; in the largest cohort assembled on this question to date, we show that its magnitude is not constant across the adult age range, with marked attenuation in the oldest patients. More recent work on VIPs has emphasized that conventional ICU-relevant prognostic markers may carry different weight in this population, consistent with the present findings [7,8]. In VIPs, each additional week in hospital before ICU admission added a smaller absolute risk increment than seen in the majority of younger deciles. Absolute mortality nevertheless remained very high in VIPs throughout the LOS range (34.6 % at day 0, 47.4 % at day 60); pre-ICU LOS in this group is therefore better understood as a weaker prognostic marker than as an unimportant one. Two non-exclusive mechanisms can explain this attenuation. A ceiling effect operates at the mortality end: when day-0 mortality already approaches one in three, the absolute risk attributable to further time in hospital has limited room to rise even though the proportional change relative to the baseline may remain substantial. A selection mechanism operates at the other end: triage to the ICU is itself age-dependent, older patients are admitted more selectively than younger ones, and VIPs who reach the ICU after a long ward stay form a survivor population pre-selected for robustness. This selection alone can flatten the apparent effect of pre-ICU LOS in VIPs, and it subsumes the more general observation that longer pre-ICU stays are accumulated by different kinds of patients at different ages.

We deliberately did not adjust for SAPS3 Box III in the primary analysis. Box III is measured at ICU admission, after the exposure window, and is a plausible mediator between pre-ICU hospital length of stay and short-term mortality: a longer pre-ICU stay may worsen physiological status at admission, which in turn raises mortality. Conditioning on a mediator blocks part of the total effect we wished to estimate and would introduce overadjustment bias (Additional file, Figure S1). We therefore added Box III only in a sensitivity analysis, to examine how much of the association persisted once acute physiology at admission was held fixed. The age-dependent pattern was preserved in that analysis. Within-age risk differences in middle-aged and older deciles showed an initial decrease in predicted 30-day mortality during the first days of pre-ICU hospitalization, followed by the expected rise. A plausible mechanism is that direct-from-ER (emergency room) admissions, who made up 51 % of the cohort, arrived with more severe acute physiological derangement on average than patients who deteriorated on a hospital ward. The Box III sensitivity analysis supports this interpretation: adjusting for SAPS3 Box III systematically reduced the magnitude of the initial dip across all affected deciles, and reversed its sign at day 7 in VIPs. The residual dip that persisted after Box III adjustment may reflect unmeasured aspects of physiological derangement.

We allowed age and sex to interact in the model because age-related increases in mortality are steeper in men than in women across adult life [26]. Our primary estimands are marginalized over the within-decile sex distribution.

The major strengths of this study are the large national cohort, with SIR coverage of Swedish general ICUs rising from approximately 50 % in 2005 to 100 % by 2017, minimal expected attrition from emigration within the 30-day window, and effectively no missingness in the primary adjustment set (age, sex, CCI and outcome complete by register design; pre-ICU LOS reconstructed from NPR when SAPS3 reporting was missing). A methodological strength is reporting absolute risks and risk differences at clinically interpretable time points rather than hazard ratios, which suffer built-in selection bias from the depletion of susceptibles and offer no advantage here given complete follow-up [27].

Several limitations apply. First, the number of patients with pre-ICU LOS beyond 30 days is limited, reflected in wider confidence intervals at the right end of Fig. 1. Second, SAPS3 Box III was missing in approximately 39 % of patients in the sensitivity analysis, handled by multiple imputation; the consistency of the primary and Box III analyses makes it unlikely that missingness drove our findings. Third, our cohort is conditioned on reaching the ICU: older patients who died on a ward during a long pre-ICU stay, or who were never admitted to the ICU despite deteriorating, do not contribute, so age-dependent selection and competing non-ICU death may bias the estimates in ways we cannot quantify Fourth, the cohort is also relatively historical, spanning 2005–2016: the very old ICU population has since shifted in baseline health, comorbidity burden, and admission practice, so contemporary ≥80-year-olds may not be directly comparable to those studied here, and the age-specific estimates are best read as describing that period rather than current practice. This window does, however, predate the COVID-19 pandemic and its disruptions to ICU triage and capacity, so it characterizes the stable age-dependent association between pre-ICU LOS and short-term mortality without confounding from a period of unusual resource pressure. Finally, residual confounding by unmeasured factors such as frailty indices not derivable from NPR or details of pre-ICU management cannot be excluded. Findings should generalize to ICU systems with similar triage traditions, primarily Scandinavian and Western European settings.

## Conclusion

The absolute incremental mortality risk attributable to each additional day of pre-ICU hospital length of stay was visibly attenuated in VIPs, compared with the majority of ICU patients, against a background of very high baseline 30-day mortality. The flatter age-specific gradient suggests that the preceding time in hospital carries less prognostic information when triaging a VIP for ICU admission than unstratified estimates from the adult ICU literature would imply, although the extent to which this reflects biology versus pre-ICU triage cannot be fully resolved from these data.

## Authors' contributions

BA conceived the study, ML acquired the data, BA and ML analyzed the data, BA wrote a first draft of the manuscript, and all authors revised the text and approved the final draft.

## Consent for publication

Not applicable.

## Ethics approval and consent to participate

The study was approved by the Regional Ethics Committee of Uppsala (DNR 2016/421) and consent was waived.

## Declaration of Generative AI and AI-assisted technologies in the writing process

During the preparation of this work the authors used Claude AI in order to check and debug R code and in Language editing of the manuscript. After using this tool, the authors reviewed and edited the content as needed and takes full responsibility for the content of the published article.

## Funding

The Akademiska University Hospital, Uppsala, Sweden and the healthcare Region Dalarna, Sweden funded this research. The funding sources had no role in the design and conduct of the study; the collection, management, analysis, or interpretation of the data; the preparation, review, or approval of the manuscript; and the decision to submit the manuscript for publication.

## Availability of data and material

The data that support the findings of this study are available from the registry holders (the Swedish Intensive Care Registry and the Swedish Board of Health and Welfare), but restrictions apply to the availability of these data, which were used under license for the current study, and so are not publicly available. Data are however available from the authors upon reasonable request and with permission of the Swedish Ethical Review Authority and Uppsala University, under the limitations of the European General Data Protection Act.

## Declaration of competing interest

The authors declare that they have no competing interests
