# Supplementary material for: Pre-ICU hospital length of stay and 30-day mortality in the very old: a national cohort of 315 042 ICU admissions
Source: Ann Intensive Care. 2026 Jul 16;16:100116. doi: 10.1016/j.aicoj.2026.100116 (PMC13396954; doi:10.1016/j.aicoj.2026.100116)
Supplement: Supplementary file 1 [file mmc1.docx]

**Pre-ICU hospital length of stay and 30-day mortality in the very old: a national cohort of 315 042 ICU admissions – Additional file**

*Björn Ahlström^1,2^, Miklos Lipcsey^1,3^

^1^Anesthesiology and Intensive Care, Department of Surgical Sciences, Uppsala University, Uppsala, Sweden.

^2^Center for Clinical Research Dalarna, Uppsala University, Sweden.

^3^Hedenstierna laboratory, Department of Surgical Sciences, Uppsala University, Uppsala, Sweden.

*Corresponding author, E-mail: [bjorn.ahlstrom@uu.se](mailto:bjorn.ahlstrom@uu.se)

Contents

[**Fig. S1** Directed acyclic graph 2](#_Toc233394032)

[**Supplementary statistical methods** 2](#_Toc233394033)

[Primary model 2](#_Toc233394034)

[Estimands and G-computation (standardization) 3](#_Toc233394035)

[Uncertainty (nonparametric bootstrap) 3](#_Toc233394036)

[Model performance 3](#_Toc233394037)

[Sensitivity analyses 3](#_Toc233394038)

[**Fig. S2** Bootstrap-corrected calibration of the primary model 4](#_Toc233394039)

[**Table S1.** Primary model performance 4](#_Toc233394040)

[**Fig. S3** Imputation diagnostic convergence trace plots 5](#_Toc233394041)

[**Fig. S4** Imputation diagnostic density plot for SAPS 3 box III 5](#_Toc233394042)

[**Table S2.** Statistical software and R packages used 6](#_Toc233394043)

[**Table S3**. Between-age risk differences 7](#_Toc233394044)

[**Fig. S5** Sensitivity analysis. Estimated probability of 30-day mortality 8](#_Toc233394045)

[**Table S4.** Sensitivity analysis. Within-age risk differences 8](#_Toc233394046)

[**Table S5.** Sensitivity analysis. Between-age risk differences 9](#_Toc233394047)

[**Fig. S6** Sensitivity analysis. Estimated probability of 30-day mortality 9](#_Toc233394048)

[**Table S6.** Sensitivity analysis. Within-age risk differences 10](#_Toc233394049)

[**Table S7.** Sensitivity analysis. Between-age risk differences 10](#_Toc233394050)

| 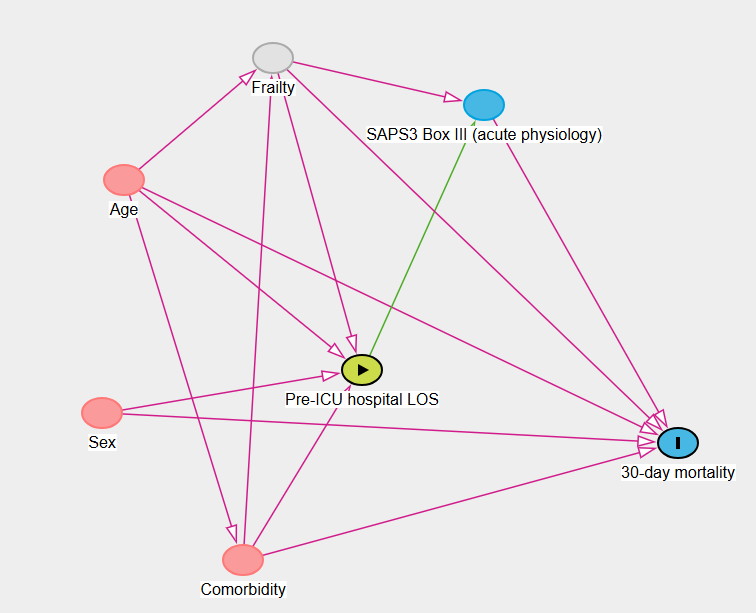 |
| --- |
| **Fig. S1** Directed acyclic graph for the assumed causal structure (1). **The graph was made using** <https://www.dagitty.net/dags.html>**.** Exposure (pre-ICU hospital length of stay) and outcome (30-day all-cause mortality from ICU admission) are highlighted. Age, sex, and Quan's updated Charlson comorbidity index are common causes of both exposure and outcome and constitute the adjustment set for the primary model (red nodes). Frailty is a plausible confounder but was not directly measurable from the registries; it is shown as unmeasured (grey node). SAPS3 Box III, representing acute physiological derangement at ICU admission, is positioned as a post-exposure mediator: a longer pre-ICU stay plausibly worsens physiology at admission, which in turn raises short-term mortality. Adjusting for SAPS3 Box III in the primary analysis would block part of the total effect of pre-ICU length of stay on mortality and introduce overadjustment bias; it was therefore examined only in a sensitivity analysis |

## **Supplementary statistical methods**

### Primary model

The primary model was a binary logistic regression for 30-day all-cause mortality, fit by maximum likelihood. Continuous predictors entered as restricted cubic splines at Harrell’s default knot percentiles: age and log(pre-ICU LOS + 1) with four knots each, and the Quan-updated Charlson comorbidity index (CCI) with three knots. Sex entered as a binary factor. Three pairwise interactions were included: age × log(pre-ICU LOS), log(pre-ICU LOS) × sex, and age × sex, each encoded as products of the corresponding spline bases (spline-by-factor for the sex interactions). The age × log(pre-ICU LOS) interaction encodes the primary estimand, the modification of the pre-ICU LOS association by age. The model was fit on all patients with complete data on the adjustment set (N = 315 042).

### Estimands and G-computation (standardization)

From the fitted model we derived three estimands at seven pre-ICU LOS values (t = 0, 1, 3, 7, 14, 28, and 60 days) by G-computation (standardization) (2). For a given age decile, the standardized (marginal) predicted risk at pre-ICU LOS t was obtained by setting log(pre-ICU LOS + 1) to log(t + 1) for every patient in that decile while holding their observed age, sex, and CCI fixed, computing each patient’s model-predicted probability, and averaging these probabilities over the decile. This averages over the decile’s observed joint distribution of age, sex, and CCI rather than over fixed covariate values. The three estimands were: (i) the standardized predicted probability of 30-day mortality for each age decile at each t; (ii) the within-age risk difference (RD), the standardized risk at t minus the standardized risk at t = 0 within the same decile; and (iii) the between-age RD, the standardized risk for a given decile minus that for the 18–29 reference decile at the same t. Each decile’s standardized risk uses that decile’s own covariate distribution, so the between-age RDs are marginal contrasts between the actual age-group populations, not counterfactual contrasts for the same patients at different ages.

### Uncertainty (nonparametric bootstrap)

Confidence intervals for all three estimands were obtained by a nonparametric bootstrap: 1000 resamples of individuals drawn with replacement, with the full logistic model refit and all estimands recomputed in each resample (3). Reported 95% CIs are the 2.5th and 97.5th percentiles of the bootstrap distribution and standard errors are the bootstrap standard deviations. The within-age and between-age RDs were computed from the same resamples — the within-age RD is the difference between an age decile’s standardized risk at t and at t = 0 within each replicate — so both sets of RDs share identical percentile-bootstrap inference.

### Model performance

Discrimination (Harrell’s C-index) and calibration of the primary model were assessed by optimism-corrected bootstrap with 1000 resamples (rms::validate and rms::calibrate). The bootstrap-corrected calibration curve and the apparent and optimism-corrected performance metrics are shown in Figure S2 and Table S1.

### Sensitivity analyses

Two sensitivity analyses were performed. In the first, continuous age and log(pre-ICU LOS + 1) were replaced by their SAPS3 Box I categorical forms (age: <40, 40–59, 60–69, 70–74, 75–79, ≥80; pre-ICU LOS: 0–13, 14–27, ≥28 days), retaining sex, CCI (restricted cubic spline, three knots), and all pairwise interactions; inference used the same nonparametric bootstrap as the primary model. In the second, SAPS3 Box III (restricted cubic spline, three knots) was added to the primary model. SAPS3 Box III was missing in approximately 39% of patients and was imputed by multivariate imputation by chained equations (MICE; 30 imputations, 10 iterations, predictive mean matching) (4). Within each imputed dataset the full G-computation pipeline was run with a nested 1000-replicate bootstrap, and point estimates and variances were pooled across imputations using Rubin’s rules adapted for G-computation estimands. MICE convergence trace plots and observed-versus-imputed diagnostics are shown in Figures S3 and S4.

| **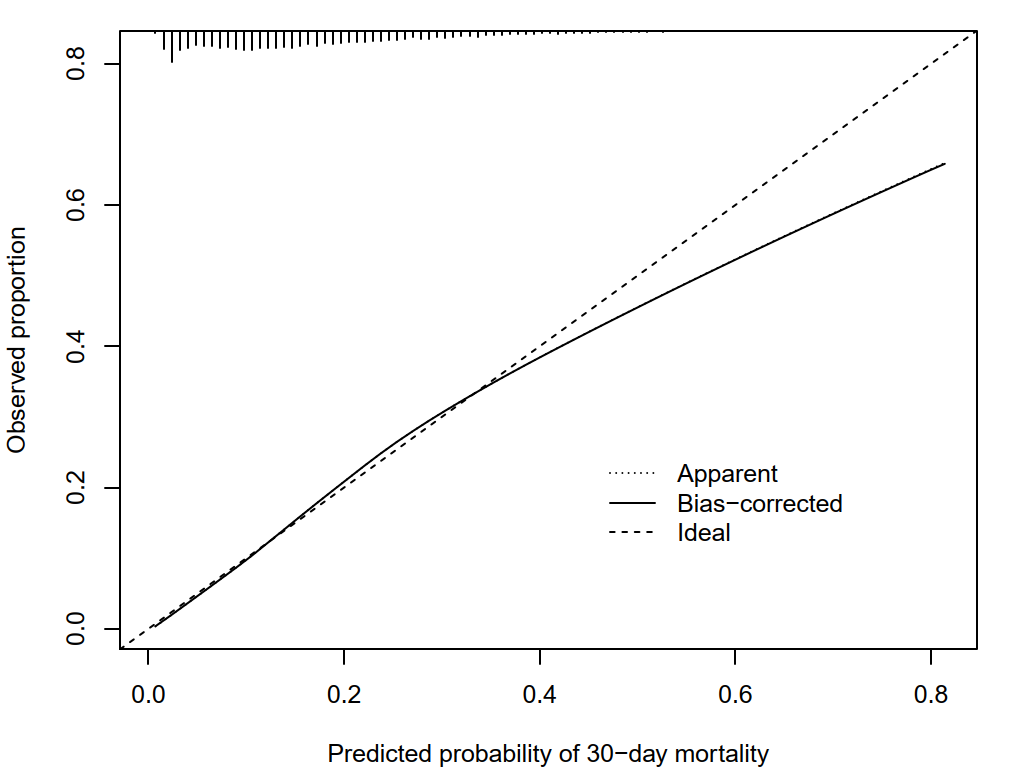** |
| --- |
| **Fig. S2** Bootstrap-corrected calibration of the primary model. Observed proportion of 30-day deaths (y-axis) against predicted probability (x-axis), smoothed nonparametrically, for the primary logistic regression on N = 315 042 patients. The bias-corrected curve is based on 1000 bootstrap resamples (rms::calibrate). The dashed diagonal represents ideal calibration. Mean absolute error between apparent and bias-corrected curves was 0.006, indicating excellent calibration across the full range of predicted risk |

| **Table S1.** Primary model performance, optimism-corrected by 1000 bootstrap resamples (rms::validate). Reports apparent and optimism-corrected values of Harrell's C-index, Somers' Dxy, Brier score, Nagelkerke R², and calibration slope. | |
| --- | --- |
| metric | value |
| C-index (apparent) | 0.740 |
| C-index (optimism-corrected) | 0.740 |
| Dxy (apparent) | 0.481 |
| Dxy (optimism-corrected) | 0.481 |
| Brier (apparent) | 0.123 |
| Brier (optimism-corrected) | 0.123 |
| R2 (apparent) | 0.164 |
| R2 (optimism-corrected) | 0.164 |
| Slope (apparent) | 1.000 |
| Slope (optimism-corrected) | 0.999 |

| 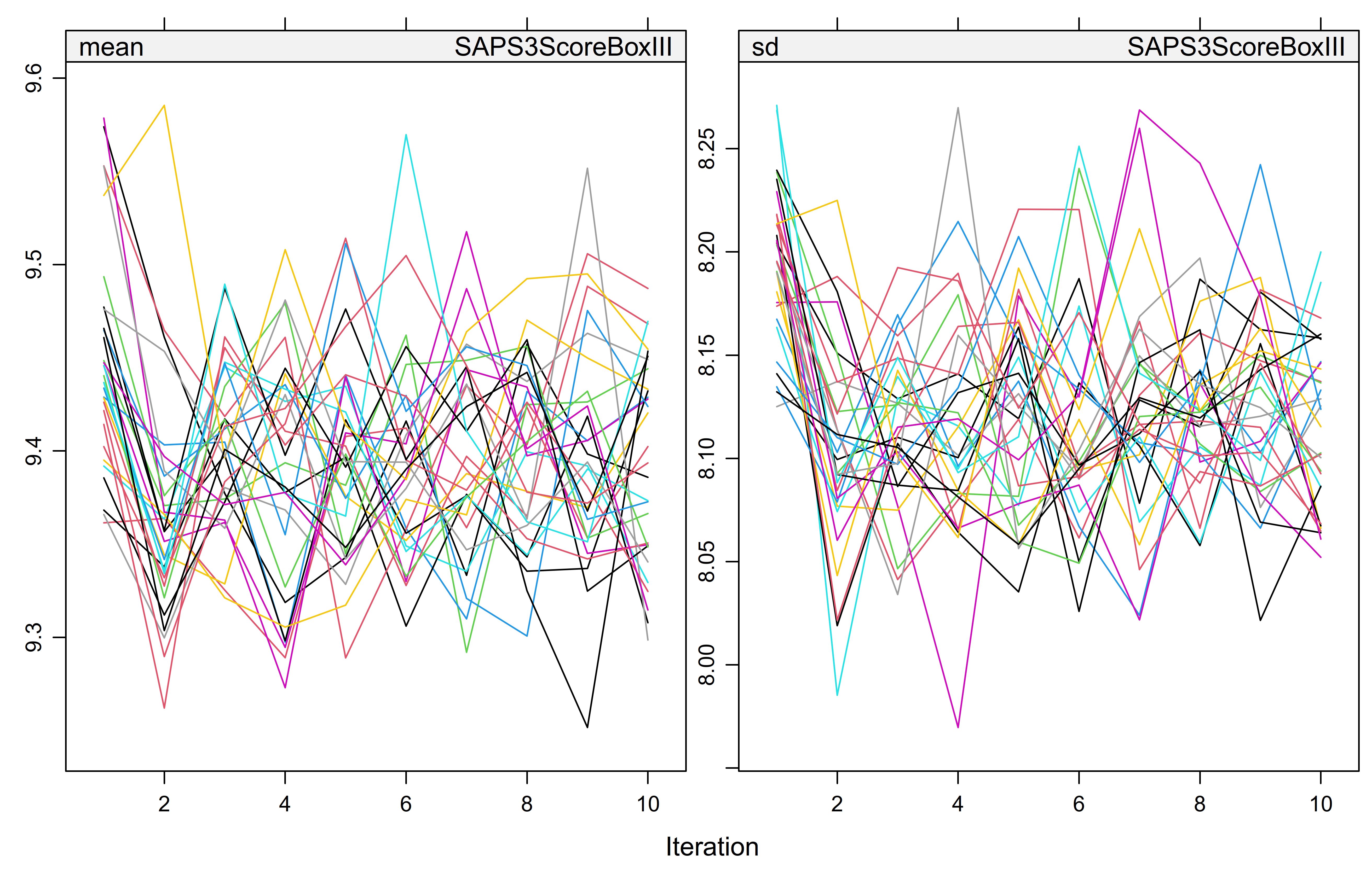 |
| --- |
| **Fig. S3** Imputation diagnostic convergence trace plots for SAPS 3 box III |

| 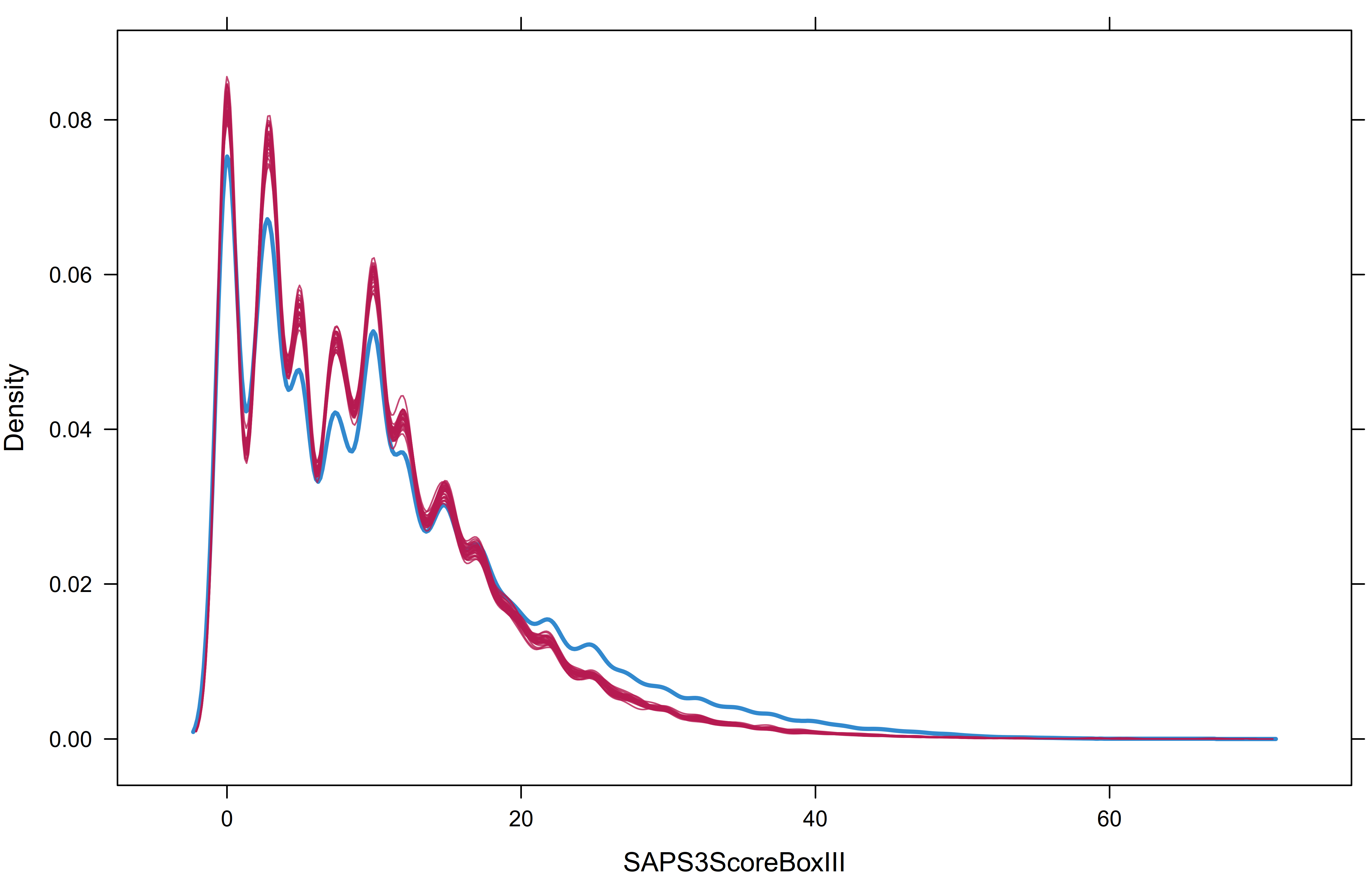 |
| --- |
| **Fig. S4** Imputation diagnostic density plot for SAPS 3 box III. Kernel density of SAPS3 Box III in observed data (thick blue line) and in each of the 30 imputed datasets (thin red lines) |

| **Table S2.** Statistical software and R packages used. | | |
| --- | --- | --- |
| **Tool** | **Citation** |  |
| R | R Core Team (2024). R: A language and environment for statistical computing. R Foundation for Statistical Computing, Vienna, Austria. https://www.R-project.org/ |  |
| tidyverse | Wickham H, Averick M, Bryan J, et al. (2019). Welcome to the tidyverse. Journal of Open Source Software 4(43):1686. doi:10.21105/joss.01686 |  |
| lubridate | Grolemund G, Wickham H (2011). Dates and Times Made Easy with lubridate. Journal of Statistical Software 40(3):1–25. doi:10.18637/jss.v040.i03 |  |
| rms | Harrell FE Jr (2024). rms: Regression Modeling Strategies. R package version 6.8-1. https://CRAN.R-project.org/package=rms |  |
| mice | van Buuren S, Groothuis-Oudshoorn K (2011). mice: Multivariate Imputation by Chained Equations in R. Journal of Statistical Software 45(3):1–67. doi:10.18637/jss.v045.i03 |  |
| FSA | Ogle DH, Doll JC, Wheeler AP, Dinno A (2024). FSA: Simple Fisheries Stock Assessment Methods. R package version 0.9.5. https://CRAN.R-project.org/package=FSA |  |
| marginaleffects | Arel-Bundock V, Greifer N, Heiss A (2024). How to Interpret Statistical Models Using marginaleffects for R and Python. Journal of Statistical Software 111(9):1–32. doi:10.18637/jss.v111.i09 |  |
| parallel | Part of base R; see R Core Team (2024), above. |  |
| readr | Wickham H, Hester J, Bryan J (2024). readr: Read Rectangular Text Data. R package version 2.1.5. https://CRAN.R-project.org/package=readr |  |
| readxl | Wickham H, Bryan J (2023). readxl: Read Excel Files. R package version 1.4.3. https://CRAN.R-project.org/package=readxl |  |
| haven | Wickham H, Miller E, Smith D (2023). haven: Import and Export 'SPSS', 'Stata' and 'SAS' Files. R package version 2.5.4. https://CRAN.R-project.org/package=haven |  |
| labelled | Larmarange J (2024). labelled: Manipulating Labelled Data. R package version 2.13.0. https://CRAN.R-project.org/package=labelled |  |
| janitor | Firke S (2023). janitor: Simple Tools for Examining and Cleaning Dirty Data. R package version 2.2.0. https://CRAN.R-project.org/package=janitor |  |
| flextable | Gohel D, Skintzos P (2024). flextable: Functions for Tabular Reporting. R package version 0.9.6. https://CRAN.R-project.org/package=flextable |  |
| officer | Gohel D, Moog S (2024). officer: Manipulation of Microsoft Word and PowerPoint Documents. R package version 0.6.6. https://CRAN.R-project.org/package=officer |  |
| openxlsx | Schauberger P, Walker A (2023). openxlsx: Read, Write and Edit xlsx Files. R package version 4.2.5.2. https://CRAN.R-project.org/package=openxlsx |  |
| gridExtra | Auguie B (2017). gridExtra: Miscellaneous Functions for 'Grid' Graphics. R package version 2.3. https://CRAN.R-project.org/package=gridExtra |  |

| **Table S3**. Between-age risk differences (percentage points) for 30-day mortality at each pre-ICU hospital length of stay, relative to the 18-29 age decile. Primary model. | | | | | | | |
| --- | --- | --- | --- | --- | --- | --- | --- |
| **Age decile** | **Day 0** | **Day 1** | **Day 3** | **Day 7** | **Day 14** | **Day 28** | **Day 60** |
| 30-39 | 1.6 (1.6 to 1.7) | 1.3 (1.2 to 1.4) | 1.4 (1.2 to 1.5) | 1.8 (1.5 to 2.0) | 2.5 (2.1 to 2.8) | 3.5 (2.8 to 4.1) | 4.9 (3.5 to 6.1) |
| 40-49 | 4.5 (4.3 to 4.8) | 3.4 (3.1 to 3.6) | 3.3 (2.9 to 3.7) | 4.3 (3.7 to 4.8) | 5.9 (5.0 to 6.7) | 8.1 (6.5 to 9.5) | 11.2 (8.3 to 13.8) |
| 50-59 | 9.0 (8.7 to 9.4) | 6.2 (5.9 to 6.6) | 5.8 (5.3 to 6.4) | 7.4 (6.6 to 8.1) | 9.9 (8.8 to 11.0) | 13.3 (11.3 to 15.2) | 18.0 (14.1 to 21.3) |
| 60-69 | 14.8 (14.5 to 15.1) | 9.8 (9.5 to 10.2) | 9.0 (8.5 to 9.5) | 11.0 (10.3 to 11.7) | 14.4 (13.3 to 15.4) | 18.8 (16.8 to 20.5) | 24.3 (20.8 to 27.3) |
| 70-79 | 22.5 (22.2 to 22.9) | 16.4 (16.0 to 16.7) | 15.3 (14.8 to 15.8) | 17.7 (17.0 to 18.3) | 21.4 (20.4 to 22.4) | 25.8 (24.0 to 27.4) | 30.8 (27.7 to 33.6) |
| 80+ | 32.9 (32.4 to 33.5) | 29.0 (28.3 to 29.6) | 28.4 (27.6 to 29.2) | 30.4 (29.5 to 31.4) | 33.0 (31.5 to 34.5) | 35.5 (33.0 to 37.8) | 37.7 (33.8 to 41.6) |
|  |  |  |  |  |  |  |  |
| Values are risk differences in percentage points with 95 % bootstrap percentile CI in parentheses. Estimates obtained by G-computation as described for Table 3. | | | | | | | |

| **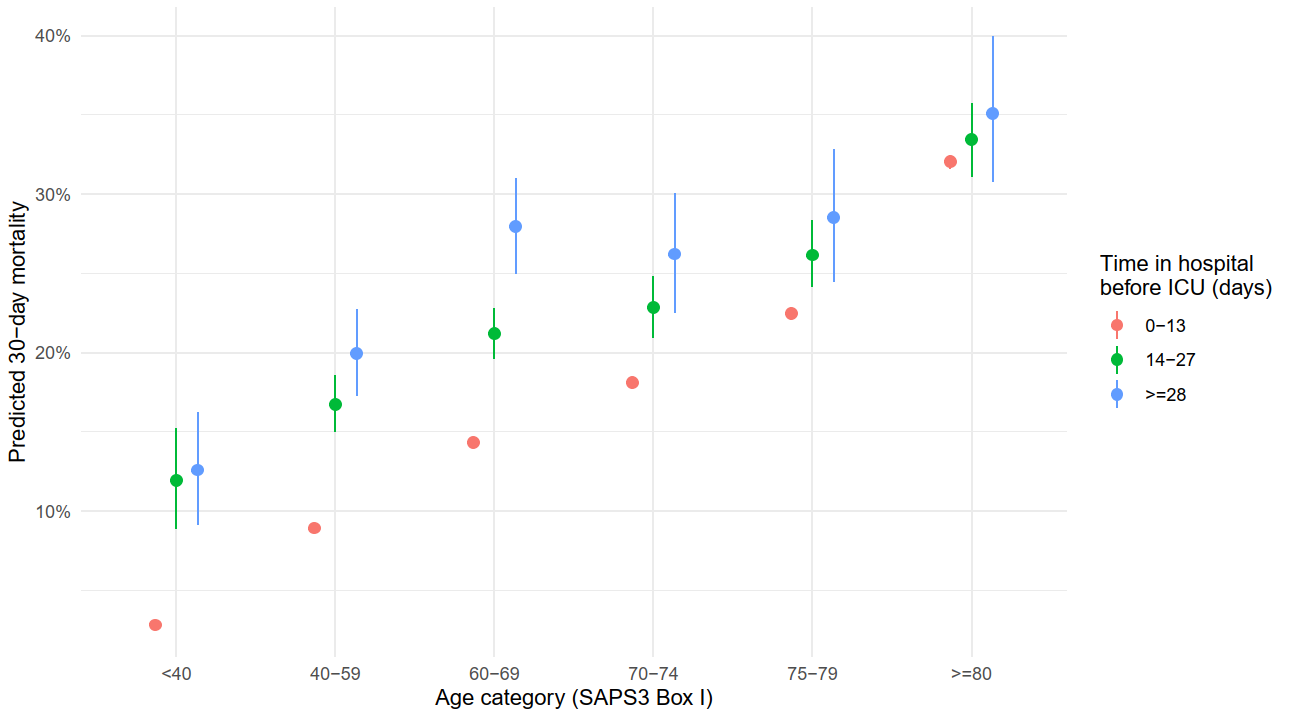** |
| --- |
| **Fig. S5** Sensitivity analysis. Estimated probability of 30-day mortality by SAPS3 age category and time in hospital before ICU admission divided into SAPS3 scoring groups. The error bars represent  the 95% CI |

|  | | |
| --- | --- | --- |
| **Table S4.** Sensitivity analysis. Within-age risk differences (percentage points) for 30-day mortality across pre-ICU hospital length of stay, relative to 0-13 days. Sensitivity analysis with SAPS3 Box I categorical forms. | | |
| **Age category (SAPS3 Box I)** | **14-27 days** | **>=28 days** |
| <40 | 7.8 (5.2 to 10.7) | 8.4 (5.4 to 11.7) |
| 40-59 | 7.5 (5.8 to 9.4) | 10.6 (8.0 to 13.3) |
| 60-69 | 6.9 (5.3 to 8.5) | 13.6 (10.7 to 16.6) |
| 70-74 | 4.9 (2.9 to 6.9) | 8.3 (4.5 to 12.1) |
| 75-79 | 3.8 (1.7 to 6.1) | 6.2 (2.0 to 10.7) |
| >=80 | 1.5 (-1.0 to 3.9) | 3.3 (-1.2 to 8.4) |
|  |  |  |
| Values are risk differences in percentage points with 95 % bootstrap percentile CI in parentheses. Age and pre-ICU LOS replaced by their SAPS3 Box I categorical forms; sex, CCI (rcs, 3 knots) and all pairwise interactions retained. | | |

| **Table S5.** Sensitivity analysis. Between-age risk differences (percentage points) for 30-day mortality at each pre-ICU hospital length of stay, relative to age <40. Sensitivity analysis with SAPS3 Box I categorical forms. | | | |
| --- | --- | --- | --- |
| **Age category (SAPS3 Box I)** | **0-13 days** | **14-27 days** | **>=28 days** |
| 40-59 | 6.2 (5.9 to 6.5) | 5.9 (2.6 to 9.1) | 8.4 (4.2 to 12.5) |
| 60-69 | 12.3 (12.0 to 12.6) | 11.4 (8.0 to 14.5) | 17.5 (13.1 to 22.0) |
| 70-74 | 16.6 (16.2 to 17.1) | 13.7 (10.4 to 17.1) | 16.5 (11.8 to 21.2) |
| 75-79 | 21.4 (21.0 to 21.9) | 17.4 (13.8 to 20.9) | 19.3 (13.6 to 24.3) |
| >=80 | 31.4 (30.9 to 31.8) | 25.1 (21.4 to 28.7) | 26.3 (20.7 to 31.8) |
|  |  |  |  |
| Values are risk differences in percentage points with 95 % bootstrap percentile CI in parentheses. | | | |

| 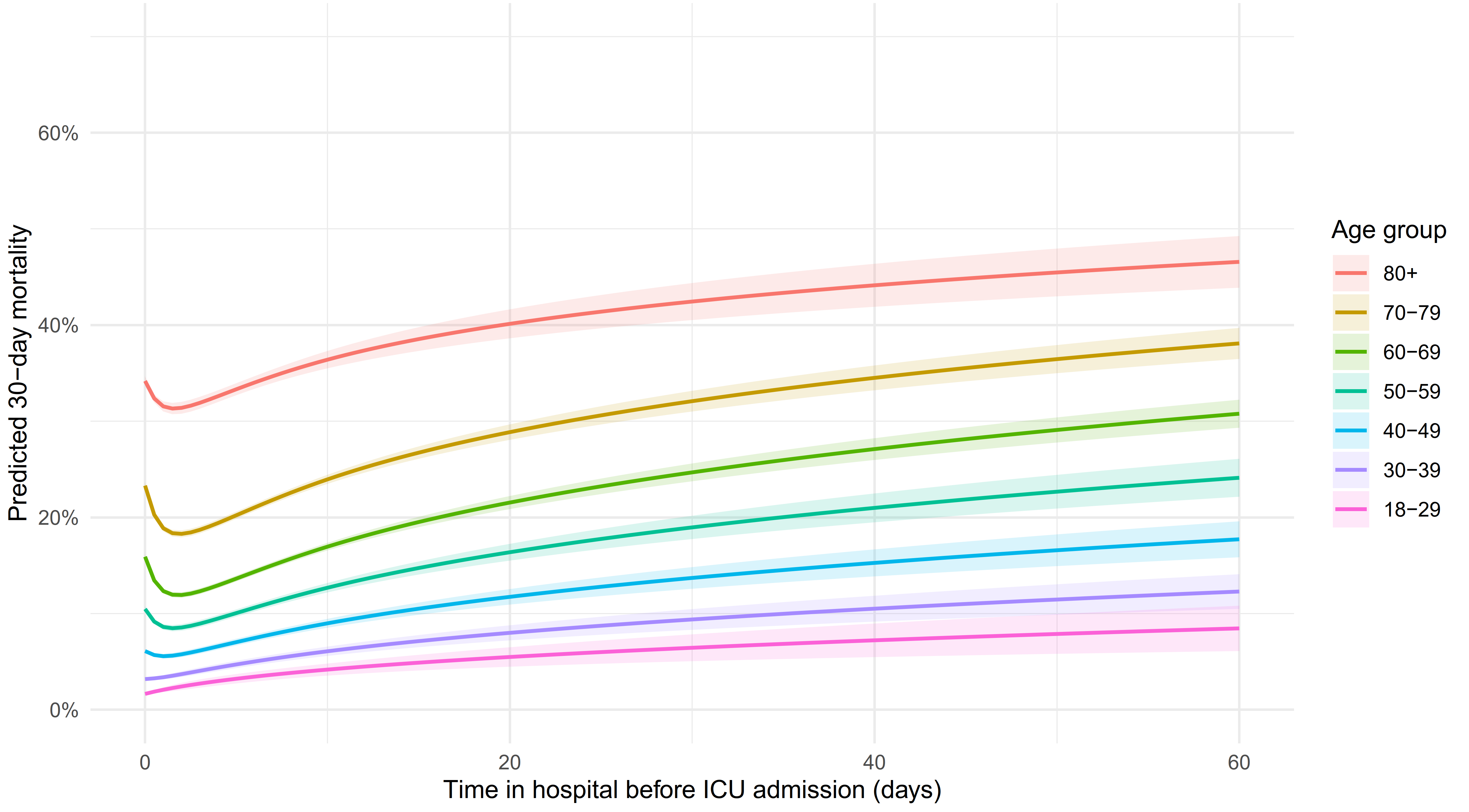 |
| --- |
| **Fig. S6** Sensitivity analysis. Estimated probability of 30-day mortality by age decile and time in hospital before ICU admission, SAPS3 box III added to the model. The shaded areas represent the 95% CI computed by the delta method |

| **Table S6.** Sensitivity analysis. Within-age risk differences (percentage points) for 30-day mortality across pre-ICU hospital length of stay, relative to day 0. Sensitivity analysis with SAPS3 Box III added to the model. | | | | | | |
| --- | --- | --- | --- | --- | --- | --- |
| **Age decile** | **Day 1** | **Day 3** | **Day 7** | **Day 14** | **Day 28** | **Day 60** |
| 18-29 | 0.4 (0.2 to 0.7) | 1.1 (0.6 to 1.5) | 2.0 (1.4 to 2.6) | 3.1 (2.3 to 3.9) | 4.6 (3.3 to 6.0) | 6.8 (4.4 to 9.2) |
| 30-39 | 0.2 (-0.0 to 0.4) | 0.9 (0.5 to 1.2) | 2.1 (1.7 to 2.6) | 3.8 (3.1 to 4.4) | 5.9 (4.9 to 7.0) | 9.1 (7.3 to 10.9) |
| 40-49 | -0.5 (-0.8 to -0.3) | 0.1 (-0.3 to 0.4) | 1.8 (1.3 to 2.2) | 4.1 (3.5 to 4.8) | 7.3 (6.2 to 8.3) | 11.6 (9.7 to 13.5) |
| 50-59 | -1.9 (-2.2 to -1.5) | -1.5 (-2.0 to -1.0) | 0.7 (0.2 to 1.2) | 3.9 (3.2 to 4.6) | 8.0 (6.8 to 9.2) | 13.6 (11.7 to 15.6) |
| 60-69 | -3.6 (-3.9 to -3.2) | -3.6 (-4.0 to -3.2) | -0.9 (-1.3 to -0.4) | 3.1 (2.6 to 3.7) | 8.2 (7.3 to 9.1) | 14.9 (13.4 to 16.3) |
| 70-79 | -4.4 (-4.9 to -4.0) | -4.6 (-5.2 to -4.0) | -1.5 (-2.1 to -0.9) | 2.9 (2.2 to 3.6) | 8.2 (7.2 to 9.3) | 14.8 (13.2 to 16.4) |
| 80+ | -2.6 (-3.3 to -2.0) | -2.3 (-3.1 to -1.4) | 0.5 (-0.4 to 1.4) | 4.0 (2.7 to 5.3) | 7.9 (5.9 to 9.8) | 12.4 (9.6 to 15.1) |
| Values are risk differences in percentage points with 95 % bootstrap percentile CI in parentheses. | | | | | | |

| **Table S7.** Sensitivity analysis. Between-age risk differences (percentage points) for 30-day mortality at each pre-ICU hospital length of stay, relative to the 18-29 age decile. Sensitivity analysis with SAPS3 Box III added to the primary model, pooled across 30 MICE imputations. | | | | | | | |
| --- | --- | --- | --- | --- | --- | --- | --- |
| **Age decile** | **Day 0** | **Day 1** | **Day 3** | **Day 7** | **Day 14** | **Day 28** | **Day 60** |
| 30-39 | 1.5 (1.4 to 1.7) | 1.3 (1.1 to 1.5) | 1.3 (1.1 to 1.6) | 1.7 (1.4 to 2.0) | 2.2 (1.7 to 2.6) | 2.9 (2.1 to 3.6) | 3.8 (2.6 to 5.1) |
| 40-49 | 4.4 (4.2 to 4.7) | 3.5 (3.2 to 3.8) | 3.4 (3.0 to 3.9) | 4.2 (3.7 to 4.8) | 5.5 (4.6 to 6.3) | 7.1 (5.6 to 8.5) | 9.3 (6.7 to 11.8) |
| 50-59 | 8.8 (8.5 to 9.2) | 6.5 (6.1 to 6.9) | 6.2 (5.6 to 6.8) | 7.5 (6.8 to 8.3) | 9.6 (8.5 to 10.7) | 12.2 (10.3 to 14.1) | 15.7 (12.4 to 19.0) |
| 60-69 | 14.3 (13.9 to 14.6) | 10.3 (9.9 to 10.6) | 9.6 (9.0 to 10.2) | 11.4 (10.7 to 12.1) | 14.3 (13.3 to 15.3) | 17.9 (16.2 to 19.6) | 22.3 (19.4 to 25.2) |
| 70-79 | 21.7 (21.3 to 22.0) | 16.8 (16.4 to 17.2) | 16.0 (15.4 to 16.5) | 18.2 (17.5 to 18.8) | 21.5 (20.5 to 22.4) | 25.3 (23.7 to 26.8) | 29.6 (26.9 to 32.3) |
| 80+ | 32.5 (32.0 to 33.1) | 29.5 (28.9 to 30.1) | 29.2 (28.4 to 30.0) | 31.0 (30.1 to 32.0) | 33.4 (31.9 to 34.9) | 35.8 (33.4 to 38.1) | 38.1 (34.4 to 41.8) |
| Values are risk differences in percentage points with 95 % CI in parentheses. Pooling as described for Table S5. | | | | | | | |

1. Textor J, van der Zander B, Gilthorpe MS, Liskiewicz M, Ellison GT. Robust causal inference using directed acyclic graphs: the R package 'dagitty'. Int J Epidemiol. 2016;45(6):1887-94.

2. Hernán MA, Robins JM. Standardization and the parametric g-formula. In: Hernán MA, Robins JM, editors. Causal Inference: What If. Boca Raton, FL: Chapman & Hall/CRC; 2020. p. 175-86.

3. Schomaker M, Heumann C. Bootstrap inference when using multiple imputation. Stat Med. 2018;37(14):2252-66.

4. Van Buuren S. Multiple imputation. In: Van Buuren S, editor. Flexible imputation of missing data. Chapman & Hall/CRC Interdisciplinary Statistics. Boca Raton: Chapman and Hall/CRC; 2018. p. 29-62.
